# Novel Insights into Autism Knowledge and Stigmatizing Attitudes Toward Mental Illness in Dutch Youth and Family Center Physicians

**DOI:** 10.1007/s10597-020-00568-w

**Published:** 2020-02-11

**Authors:** Maarten van ‘t Hof, Ina van Berckelaer-Onnes, Mathijs Deen, Monique C. Neukerk, Rienke Bannink, Amy M. Daniels, Hans W. Hoek, Wietske A. Ester

**Affiliations:** 1 Sarr Expert Centre for Autism, Lucertis Child and Adolescent Psychiatry, Carnissesingel 51, 3083 JA Rotterdam, The Netherlands; 2 Parnassia Psychiatric Institute, Kiwistraat 30, 2552 DH The Hague, The Netherlands; 3 Faculty of Social and Behavioural Sciences, Clinical Child and Adolescent Studies, Leiden University, Wassenaarseweg 52, 2333 AK Leiden, The Netherlands; 4 Faculty of Social and Behavioural Sciences, Institute of Psychology, Methodology and Statistics Unit, Leiden University, Wassenaarseweg 52, 2333 AK Leiden, The Netherlands; 5 Department of Youth Health Care, Regional Public Health Service Rijnmond, 3003 AB Rotterdam, The Netherlands; 6 Simons Foundation, 160 Fifth Avenue, New York, NY 10010 USA; 7 Department of Psychiatry, University Medical Center Groningen, University of Groningen, Hanzeplein 1, 9713 GZ Groningen, The Netherlands; 8 Department of Epidemiology, Mailman School of Public Health, Columbia University, 722 West 168th St., New York, NY 10032 USA; 9 Department of Child and Adolescent Psychiatry, Curium-LUMC, Leiden University, Endegeesterstraatweg 27, 2342 AK Oegstgeest, The Netherlands

**Keywords:** Autism, Knowledge, Screening, Stigmatizing attitudes, Children, Physicians

## Abstract

Professionals’ limited knowledge on mental health and their stigmatizing attitudes toward mental illness can delay the diagnosis of autism. We evaluated the knowledge on Autism Spectrum Disorder (ASD) and stigmatizing attitudes in 93 physicians at Dutch Youth and Family Centers (YFC). These physicians screen for psychiatric symptoms in children. We show that their general ASD knowledge scored 7.1 (SD 1.2), but their specific ASD knowledge was only 5.7 (SD 1.7) (weighted means on 1–10 scale, 1 = least knowledge, 10 = most knowledge). Our physicians had positive attitudes toward mental illness (CAMI scores 2.18 (SD 0.33) to 2.22 (SD 0.40) on a 5-point Likert scale) but they had higher levels of stigmatizing attitudes than other Western healthcare professionals. Their levels were considerably lower than in non-Western professionals. We found no relations between ASD knowledge, stigmatizing attitudes and demographic variables. In conclusion, ASD knowledge and stigmatizing attitudes toward mental illness in Dutch YFC physicians require attention.

**Electronic supplementary material:**

The online version of this article (10.1007/s10597-020-00568-w) contains supplementary material, which is available to authorized users.

## Introduction

Autism spectrum disorders (ASD), as described in the DSM-5 (American Psychiatric Association 2013), are a serious global neurodevelopmental disorder with an estimated prevalence between 1 in every 59 to 132 people, with 52 million cases worldwide and 7.7 million disability adjusted life years (Baio et al. 2018; Baird et al. 2006; Baxter et al. 2014). An ASD is characterized by persistent deficiencies in communication and social interaction, and restricted, repetitive patterns of behaviors, interests or activities. Although most symptoms are present from early childhood, some may only manifest later in life (American Psychiatric Association 2013). While an ASD diagnosis can be established as early as 24 months (Johnson and Myers 2007), the global mean age of an ASD diagnosis is considerably later, namely between 38 and 120 months (Daniels and Mandell 2014). With an average age at diagnosis of 56–116 months in Dutch children ≤ 18 years (Begeer et al. 2013), the Netherlands is in the upper part of this global range. Where primary care is an initial approach to a medical practitioner or clinic for advice or treatment, in preventive healthcare the focus is on screening and vaccination. Preventive care medicine, as provided by physicians in Youth and Family Centers (YFC) in the Netherlands, has an important role in the early detection of ASD (Van Berckelaer-Onnes et al. 2015). Early detection and treatment are important factors to optimize development and improve lifetime outcomes for people with ASD on ASD related deficiencies like social, language and adaptive behavior kills (Fein et al. 2017; Klin et al. 2015).

YFCs provide free preventive child healthcare in all municipalities of the Netherlands and are accessible for all parents and children, regardless of their citizenship status. Parents and children can contact or visit their local YFCs, which are present in all communities regardless of the community income level. Parents can also enter the YFCs with questions regarding parenting or child development. The services of the YFCs comprise regular consultations until age 18 years, consisting of immunizations and detecting health problems and developmental delay. In the first 18 years of life, all children are invited to attend 13 individual-, 6 collective-, and 3 optional individual consultations (program varies slightly by YFC center). The individual consultations focus on medical and developmental screening, while the collective program offers vaccinations and information programs (CJG Rijnmond 2016a). YFCs, organized by municipality, strive to reach 100% of the children in The Netherlands until the age of 18. The non-response policy starts one week after two no shows on a consultation, without a message and without contact with the parents and child or adolescent. The non-response policy includes five attempts to achieve contact by phone and two attempts to perform house visits. The police are contacted when there is suspicion of a life-threatening situation. The non-response policy also includes contacting chain parties (e.g. school, youth services) and external parties (e.g. general practitioner, child day-care) to get in contact with the parent or child. The latter account also applies when the YFCs sent invitations for their preventive consultations.

One of the larger centers, the Youth and Family Center Rotterdam, offered preventive healthcare to 254,424 children in 2016 and thereby reached 98.9% of 0–4 year olds and 97.9% of 4–19 year olds (CJG Rijnmond 2016b). The municipality Rotterdam is the second largest municipality in the Netherlands with around 644.393 residents and includes the city of Rotterdam and several smaller cities (Municipality Rotterdam 2019). 53.3% of the residents hold an (first or second generation) immigration background (40% non-Western, 13.3% Western) from which the largest number of people originate (first or second generation) from Suriname (8.1%), Turkey (7.4%), Morocco (7.0%) and the Netherlands Antilles (3.9%). The municipality Rotterdam has an unemployment rate of 8.1% and holds 15.0% low income households (Municipality Rotterdam 2019). In total, the Netherlands has an unemployment rate of 4.9% and holds 7.9% low income households (CBS 2019). The YFC Rotterdam Municipality holds 23 offices in the city of Rotterdam and 39 offices in the rest of the municipality where parents and children can visit for consultation. Accessibility and services of the YFC are equal amongst all (low-, middle- and high income) districts in the municipality (CJG 2019).

There are few studies evaluating the level of ASD knowledge in physicians screening children in the general population (Online Appendix 1; Harrison et al. 2017b). Previous research indicated that the level of knowledge on ASD varies among primary care providers in the United States (Dosreis et al. 2006; Heidgerken et al. 2005). A Dutch study showed that training Dutch preventive care workers (including YFC physicians) on the early signs of ASD, ASD screening tools and protocols had a positive effect on its early detection, referral and diagnosis (Oosterling et al. 2010). However, the level of ASD knowledge was not explicitly examined in that study. In summary, there is a lack of research on physicians’ level of knowledge regarding ASD screening for mental disorders in the general population.

Stigmatizing beliefs of healthcare professionals toward mental illness have a negative effect on the help that people with mental disorders may seek (Ahmedani 2011; Almanzar et al. 2014) and can lead to patients feeling ‘labeled’ and ‘marginalized’(Liggins and Hatcher 2005). Up to 30–50% of psychiatric patients feel discriminated or stigmatized by their general practitioners (GPs) (Adriaensen et al. 2011). Stigma is defined as ‘stereotypes or negative views attributed to a person or groups of people when their characteristics or behaviors are viewed as different from or inferior to societal norms’ (Dudley 2000) and can be social stigma, self-stigma, or professional stigma (Ahmedani 2011). Stigma can be felt by the person with a mental disorder as well by their family members. Similar to other disorders or disabilities, parents of children with ASD experience stigmatization by others (Gray 1993, 2002). Studies in attention deficit hyperactivity disorder have shown that high levels of stigma in physicians who are crucial in identifying and referring these patients to psychiatric care have negative effects on their care (Tatlow-Golden et al. 2016) and dementia (Cahill et al. 2008).

Research on stigmatizing attitudes toward mental illness held by healthcare professionals such as nurses and psychiatrists found conflicting results on the association between stigmatizing attitudes toward mental illness and demographic features such as healthcare professionals’ age (Chambers et al. 2010; Hansson et al. 2013; Högberg et al. 2012; Kopera et al. 2015; Mosaku and Wallymahmed 2016; Reavley et al. 2014; Siqueira et al. 2017; Smith and Cashwell 2010; Tay et al. 2004; Vibha et al. 2008; Winkler et al. 2016) or work experience (Gras et al. 2015; Mosaku and Wallymahmed 2016; Smith and Cashwell 2010). Although stigmatizing attitudes toward ASD are often implicitly imbedded in ASD knowledge questionnaires (Harrison et al. 2017a), we found no studies evaluating ASD stigma in healthcare professionals nor any literature on how stigma toward mental illness relates to stigma toward autism. Nor did we find any studies evaluating the level of stigmatizing attitudes toward mental illness in physicians who screen for health problems and developmental issues in children in the general population.

We therefore set out to investigate the level of ASD knowledge and stigmatizing attitudes toward mental illness in Dutch YFC physicians. We also evaluated the association between ASD knowledge, stigmatizing attitudes and physician-related demographic variables. In addition, we compared the level of stigmatizing attitudes in Dutch YFC physicians with healthcare professionals in other countries.

## Methods

### Study Design and Population

We present the baseline measurements of the Dutch Live Online Learning^1^ intervention study called *Detection of Autism Spectrum Disorders in Children Aged 4–6 Years by Youth & Family Center Physicians*. The intervention was developed as part of the “Reach-Aut Academic Center for Autism; Transitions in Education” project. The intervention was performed from January through November 2016 by 93 physicians of the YFC in the Greater Rotterdam area. It consisted of three online educational meetings that were supervised by child and adolescent psychiatrists, who addressed: (1) general information about ASD and its early detection, (2) early signs of ASD and early detection during consultation, and (3) communication and referral. Each session was preceded by homework consisting of background information on ASD and clinical assignments.

### Procedure

The online course was obligatory for 93 YFC physicians in the Greater Rotterdam area. Groups of eight to eleven physicians started the intervention study each month between January and November 2016. A few days before the start of the study, we invited the YFC physicians to a test session on the online learning environment. They were asked to complete 3 questionnaires (described below) at the end of this session. The day before the start of the intervention study, a member of our research team checked whether the physicians had completed the questionnaires. If not, they were asked to complete the questionnaire at the beginning of the first intervention session.

### Measures

#### ASD Knowledge

We developed a two-part questionnaire, the Autism Spectrum Disorder Knowledge Questionnaire—physicians’ edition (AKQ-P)^2^ (Online Resource 1), specifically for our study. The first part covers 20 multiple-choice questions (Cronbach’s alpha = 0.24) on ASD general knowledge, prevalence, sex differences, and risk factors. The second part has 12 physician-specific, multiple-choice questions (Cronbach’s alpha = 0.30) that assess ASD early signs, detection, diagnostic criteria, and comorbidity. We calculated a general ASD knowledge score and a specific ASD knowledge score on a 1–10 scale (1 = least knowledge, 10 = most knowledge) using weighted item scores to account for the number of possible answers of each multiple-choice question. We calculated the weighted sum score, to correct for guessing, for the general knowledge and physician-specific sections by the following procedure. Per part of questionnaire: (1) score category (grouped by possible answers to question, six categories) = 1 + (number of questions correct in category – number of questions in category/number of possible answers) × [9/(number of questions in category – number of questions in category/number of possible answers)]. (2) Final score (score category one × number of questions in category one + score category two × number of questions in category two … score category six × number of questions in category six/total number of questions in that part of questionnaire).

The AKQ-P was evaluated and revised in three stages. First, an expert panel of 25 psychologists and one social worker with experience in working with children with ASD answered and reviewed the questionnaire during a group meeting. Then this was repeated by 24 YFC physicians from the Dordrecht region in the Netherlands. Finally, the questionnaire was tested during a pilot intervention by five of the ten YFC physicians who had participated in the course’s development. All these physicians were not part of the intervention.

#### Stigmatizing Attitudes Toward Mental Illness

We assessed stigmatizing attitudes toward mental illness using the Dutch translation of the Community Attitudes to Mental Illness (CAMI) questionnaire (Taylor and Dear 1981; Van Gampelaere 2013). This is a 40-item, self-reported questionnaire to measure attitudes toward individuals with mental illness. The internal consistency of the four CAMI scales in our sample were: authoritarianism (α = 0.60), benevolence (α = 0.70), social restrictiveness (α = 0.74) and community mental health ideology (α = 0.80). Each CAMI scale contains 10 statements scored on a 5-point Likert scale (1 = strongly agree, to 5 = strongly disagree). A value is assigned to each item and five of the 10 items for each scale are reverse coded. In order to calculate the final CAMI score, we recoded items so that a higher scale score (sum of all items) would correspond to having a more negative attitude toward people with mental illness. Responses to items in a scale were added together to obtain a score between 10 and 50 for each aspect (authoritarianism, benevolence, social restrictiveness and community mental health ideology). The scores were then divided by ten (number of items in each scale). Although the CAMI was developed to evaluate public attitudes toward mental illness (Taylor and Dear 1981), it has since been used broadly by health professionals (Chambers et al. 2010; Smith and Cashwell 2010) and others (Bell and Palmer-Conn 2018; Losinski et al. 2015).

#### International Comparison of CAMI Results

**Fig. 1:**
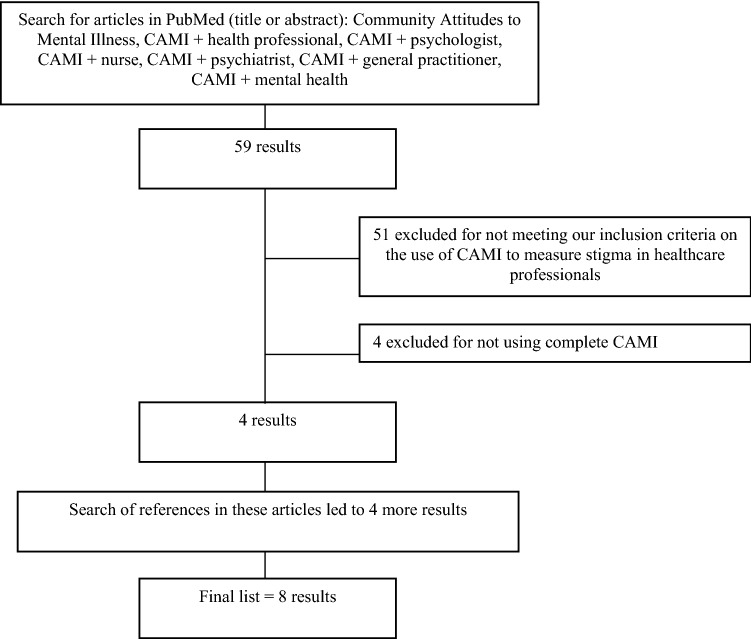
Flow diagram of literature search in PubMed to identify reports in which CAMI was used to assess stigmatizing attitudes toward mental illness held by mental healthcare professionals

To be able to compare the CAMI results of the 93 YFC physicians with international studies, we performed a systematic literature search in PubMed for studies that assessed stigma toward mental illness in mental health- and general health professionals using the CAMI (see Fig. 1 for search terms).

#### Demographic Measures

We collected demographic information from the YFC physicians by questionnaires integrated into the start of the online intervention. These covered: physician’s age, sex, ethnic background, years of work experience as a physician, and work location. Ethnic background was defined as other-Western or other non-Western if the participant, or one of the parents, was not born in the Netherlands. From the work location we were able to determine the physician’s income level using the average family income level based on data for that location provided by the municipality of Rotterdam (De Graaf 2012). The number of households per location ranged from 5610 to 43,500 for suburban regions versus urban regions. The average family income level was based on data from 2012 in euros per month and standardized for the number of family members.

### Statistical Analyses

To describe the level of ASD knowledge and stigmatizing attitudes toward mental illness held by the 93 physicians, we calculated mean scores for general ASD knowledge, specific ASD knowledge, and the CAMI scales. To evaluate the relationship between ASD knowledge, stigmatizing attitudes toward mental illness, and demographic variables, we examined the correlation between ASD knowledge, the four CAMI scale scores, age, years of experience, and income level by performing unadjusted Pearson correlations for normally distributed variables and unadjusted Spearman correlations for non-normally distributed variables. To investigate correlations found to be significant between ASD knowledge and the CAMI scale scores, we performed multiple regression analyses while controlling for sex, age, experience, general ASD knowledge score and income level. To evaluate the differences between our CAMI scale scores and those found in other studies, we re-pooled the CAMI scale scores from previous studies so that a higher CAMI score indicated more stigma. Next, we calculated the effect size, Cohen’s *d* (*d* = (M_1_ – M_2_)/S_pooled_) for all the comparable CAMI scale scores. A positive Cohen’s *d* value indicates that a study had a lower CAMI scale score than the score we had in our cohort. Cohen’s *d* represents a sizable difference if the 95% CI does not include zero, while effect sizes can be interpreted as small (0.20–0.49), medium (0.50–0.79) or large (≥ 0.80) (Cohen 1992).

### Ethical Clearance

All participants provided written informed consent before taking part in our study. The Medical Ethics Committee of Leiden University Medical Center approved the study, classifying it as not falling under the Dutch Act on Medical Research Involving Human Subjects (WMO) (ref. P15.131). All authors declared that they have no conflict of interest and certify their responsibility.

## Results

### Sample Characteristics

Sample characteristics are presented in Table 1, which has limited missing data on income level (7.6%) and on number of years’ work experience (3.2%).

**Table 1:** Sample characteristics of 93 Dutch Youth and Family Center physicians

	Percentage or median^a^	% missing
Sex		0.0
Female (%)	95.7	
Age (years)	42.0 (24.0–66.0)	0.0
Years of work experience (years)	11.0 (0.0–40.0)	3.2
Ethnicity		0.0
Dutch (%)	65.6	
Other Western (%)	15.1	
Other non-Western (%)	19.4	
Work-location-related income level	23,500 (18,500–28,700)	7.5
Below national mean^b^ (%)	70.9	
Above national mean^b^ (%)	29.1	

^a^Values are percentage for categorical variables and medians (range) for continuous non-normal distributed variables

^b^Mean standardized income level of the Netherlands is €24,200 per year

### ASD Knowledge

Our results show that general ASD knowledge was 7.1 (SD 1.2), but specific ASD knowledge was less at 5.7 (SD 1.7) (weighted means on 1–10 scale, 1 = least knowledge, 10 = most knowledge). A minority (9.7%) of the YFC physicians scored poorly on general ASD knowledge (less than 5.5, which was equivalent to answering 50% of the questions correctly) and a much larger group (41.9%) scored poorly on specific ASD knowledge. Table 2 shows the five questions topics most often answered incorrectly in the general and specific ASD knowledge part of the questionnaire.

**Table 2:** The five question topics most often answered incorrectly in the ASD knowledge questionnaire

ASD knowledge questionnaire part	Question topic	% incorrect
General ASD knowledge	ASD diagnoses in different ethnic and income groups	68
	Risk factors for developing autism	48
	The need for social contact in children with ASD	44
	Prevalence of ASD	40
	The link between ASD and hereditary and environmental factors	39
Specific ASD knowledge	The specification of Autism Spectrum Disorder in the DSM-5	67
	Syndromes in ASD	66
	Language speech and communication problems in people with ASD	64
	Possible early signs of ASD	60
	Comorbidity in ASD	54

#### Stigmatizing Attitudes Toward Mental Illness

Table 3 shows the mean (SD) CAMI scale scores for 93 YFC physicians in our study. With values below 3.00, the mean scores reveal that they have positive attitudes toward mental illness on all four CAMI scales.

**Table 3:** Mean CAMI scores, comparing the current study with previous studies in mental health and healthcare professionals

	Population (profession, country, number)	CAMI scales^a,b^			
		Authoritarianism	Benevolence	Social restrictiveness	Community mental health ideology
		Mean (SD)	Mean (SD)	Mean (SD)	Mean (SD)
Current study	YFC physicians, the Netherlands, N = 93	2.18 (.33)	2.21 (.35)	2.18 (.39)	2.22 (.40)
Smith and Cashwell (2010)	Mental health professionals, United States, N = 76	2.06 (.41)	1.69^c^ (.38)	1.89 (.42)	2.22^c^ (.50)
Chambers et al. (2010)	Mental health nurses				
	Lithuania, N = 258	2.50 (.47)	2.32^c^ (.49)	2.47 (.45)	2.47^c^ (.47)
	Italy, N = 134	2.21 (.48)	2.03^c^ (.42)	2.10 (.46)	2.06^c^ (.54)
	Ireland, N = 115	2.00 (.48)	1.85^c^ (.51)	2.00 (.47)	2.07^c^ (.59)
	Portugal, N = 125	1.96 (.43)	1.89^c^ (.43)	1.72 (.41)	1.79^c^ (.52)
	Finland, N = 178	2.10 (.37)	2.02^c^ (.42)	1.97 (.44)	2.28^c^ (.54)
Linden & Kavanagh (2012)	Mental health nurses, Ireland				
	Inpatient setting, N = 68	1.88 (.44)	1.70^c^ (.33)	1.80 (.55)	1.65^c^ (.41)
	Community setting, N = 32	1.70 (.42)	1.67^c^ (.32)	1.56 (.36)	1.45^c^ (.39)
Pitkänen et al. (2015)	Nurses psychiatric ward, Finland, N = 107	2.19 (.42)	2.06^c^ (.36)	2.05 (.47)	2.31^c^ (.59)
Siqueira et al. (2017)	Healthcare professionals, Brazil, N = 226	3.49^c^ (.47)	2.84^c^ (.44)	3.02^c^ (.50)	3.31^c^ (.52)
Mosaku and Wallymahmed (2016)	Primary care workers, Nigeria, N = 100	2.75^c^ (.50)	3.53^c^ (.52)	3.04^c^ (.65)	3.12^c^ (.23)
Al‑Awadhi et al. (2017)	Nurses, Kuwait, N = 308	2.85 (.38)	2.34^c^ (.46)	2.97 (.39)	2.52^c^ (.43)
Ebrahimi et al. (2017)	Nurses psychiatric ward, Iran, N = 93	2.60 (.33)	2.48 (.39)	2.59 (.48)	2.60 (.46)
	Nurses non-psychiatric ward, Iran, N = 105	2.63 (.36)	2.64 (.26)	2.65 (.36)	2.59 (.41)

^a^Higher scores reflect a higher level of stigma or more negative attitudes toward individuals with mental illness (range 1.00 to 5.00)

^b^Scores range from 1.00 to 5.00

^c^Repooled score for comparison

### Relationship Between ASD Knowledge, Stigmatizing Attitudes and Demographic Factors

Table 4 shows the unadjusted correlations between physicians’ ASD knowledge, stigmatizing attitudes toward mental illness, age, experience and income level. Specific ASD knowledge correlates with lower levels of authoritarian attitudes regarding people with mental illness (r(90) = − 0.208, *p* < 0.05), and higher levels of benevolent attitudes toward people with mental illness (r(90) = 0.220, *p* < 0.05). We found no correlations between specific ASD knowledge, the CAMI’s social restrictiveness and community mental health ideology scales, age, experience and income level (*p* > 0.05). Nor were any correlations found between general ASD knowledge and these factors (*p* > 0.05).

**Table 4:** Unadjusted correlations between physicians ASD knowledge, stigma and demographic measures

	1	2	3	4	5	6	7	8	9
1. General ASD knowledge score	–	.22*	− .02	− .09	− .09	− .12	− .04	− .05	.02
2. Specific ASD knowledge score		–	− .21*	− .22*	− .11	− .12	− .00	− .03	− .17
3. Stigma, authoritarianism scale^a^			–	.57**	.64**	.61**	− .02	.03	.02
4. Stigma, benevolence scale^a^				–	.46**	.54**	− .07	− .13	.04
5. Stigma, social restrictiveness scale^a^					–	.60**	.15	.08	.05
6. Stigma, community mental health ideology scale^a^						–	− .14	− .13	.04
7. Age^b^							–	.94**	.18
8. Work experience^b^								–	.22
9. Work-area-related income level^b^									–

Adjusted regressions were also performed but these were not significant

*p-value ≤ .05

**p-value ≤ .01

^a^Assessed with community attitudes to mental illness (CAMI) questionnaire

^b^Spearman rank correlation coefficients for non-normally distributed variables

To further evaluate the correlations we did find, we performed linear regression analyses, and could show that specific ASD knowledge was not associated with the authoritarianism score (F(6, 75) = 0.361, *p* = 0.901); the linear regression accounted for 2.8% of the explained variability. In adjusted analyses for sex, age, experience, and income level, the physicians’ general ASD knowledge score and ethnicity were entered into our regression, resulting in a non-significant prediction model (F(7, 74) = 0.719, *p* = 0.656).

To evaluate the CAMI’s benevolence scores, a second linear regression was performed. First, specific ASD knowledge score was entered, revealing that a higher specific ASD knowledge score was associated with a more positive benevolence attitude (F(6, 75) = 0.752, *p* = 0.610) and accounted for 5.7% of the explained variability. In adjusted analyses for sex, age, experience, and income level, the physicians’ general ASD knowledge score and ethnicity were entered into the model, resulting in a non-significant prediction model (F(7, 74) = 1.076, *p* = 0.388).

Thus, our analyses found no relationships between general or specific ASD knowledge, stigmatizing attitudes toward mental illness, age, experience, or income level in the 93 YFC physicians.

### International Comparison of CAMI Results

**Fig. 2:**
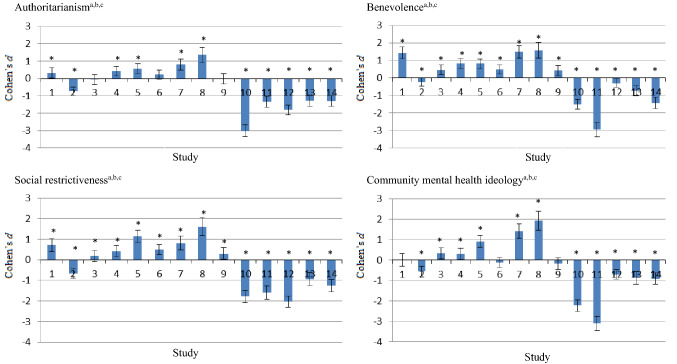
Community Attitudes to Mental Illness (CAMI) scale scores compared to literature reports using Cohen’s *d,* including 95% confidence intervals. ^a^1 = United States (Smith and Cashwell 2010), 2 = Lithuania (Chambers et al. 2010), 3 = Italy (Chambers et al. 2010), 4 = Ireland (Chambers et al. 2010), 5 = Portugal (Chambers et al. 2010), 6 = Finland (Chambers et al. 2010), 7 = Ireland-in patients (Linden and Kavanagh 2012), 8 = Ireland-community (Linden and Kavanagh 2012), 9 = Finland (Pitkänen et al. 2015), 10 = Brazil (Siqueira et al. 2017), 11 = Nigeria (Mosaku and Wallymahmed 2016), 12 = Kuwait (Al-Awadhi et al. 2017), 13 = Iran-psychiatric ward (Ebrahimi et al. 2017), 14 = Iran-non psychiatric ward (Ebrahimi et al. 2017). ^b^Positive Cohen’s *d* = lower level of psychiatric stigma than that shown by our 93 YFC physicians, and negative Cohen’s *d* = higher level of stigma. ^c^Cohen’s *d*: small (0.20–0.49), medium (0.50–0.79) or large (≥ 0.80) effect. *Different CAMI scale score than in our current study (Cohen’s *d* 95% CI does not include 0.0)

Figure 1 shows the results of our literature search. We found eight studies that reported mean CAMI scores for mental health- or other healthcare professionals. Table 2 compares the mean scores on the CAMI scales of our physicians and those of the eight literature reports. Figure 2 compares our CAMI scale scores with those from the eight studies using Cohen’s *d.* On 26/36 scales, the Dutch YFC physicians showed higher levels of stigmatizing attitudes toward mental illness than those seen in other Western healthcare professionals. However, on 20 scales that were compared with scores of non-Western professionals, Dutch YFC physicians had lower levels of stigmatizing attitudes.

## Discussion

We show that Dutch YFC physicians have sufficient general knowledge on ASD, but that a considerable number of them scored less well on specific ASD knowledge. They generally hold positive attitudes toward mental illness, but do show higher levels of stigmatizing attitudes than other Western healthcare professionals. They have lower stigmatizing attitude than non-Western professionals. We found no relationship between the level of ASD knowledge, stigmatizing attitudes toward mental illness and physician-related demographic factors.

Specific autism knowledge, as measured by our knowledge questionnaire, was insufficient in 41.9% of them. These YFC physicians play a vital role in identifying ASD symptoms in children in the Netherlands as they screen around 95% of them between the ages of two weeks and four years (GGD Nederland 2010) and follow them through childhood up to 18 years of age. Although it is widely assumed that a higher level of ASD knowledge in preventive care providers should be associated with enhanced or earlier detection of ASD, we only know of a few studies on this subject. Two studies reported a positive effect on the child’s age at diagnosis from implementing early detection strategies in preventive care providers (Chakraharti et al. 2005; Oosterling et al. 2010), but these studies did not evaluate the providers’ actual level of ASD knowledge. We found one pilot intervention study that evaluated ASD knowledge in primary care providers (pediatricians and general practitioners), which showed that a higher level of knowledge was related to more suspected cases of ASD being referred to specialists (Bordini et al. 2015).

Besides providing knowledge about the early signs of autism, the Dutch ASD guideline also provides other essential elements to help improve the early detection of ASD, namely information on autism screening tools and parent communication, and more information regarding local referral options and procedures (Van Berckelaer-Onnes et al. 2015). Thus, although we evaluated multiple aspects of ASD knowledge in our questionnaire, the physicians’ level of ASD knowledge may not fully represent their ability to detect and recognize the early signs of ASD during a consultation. So, although our study shows that a considerable group of the YFC physicians have insufficient specific ASD knowledge, more research is needed to evaluate how this might affect the early detection of ASD cases.

Our results further show that YFC physicians hold positive attitudes toward mental illness, but the levels of stigmatizing attitudes are mostly higher than those found in other Western professionals, although lower than those in non-Western professionals. We found one study evaluating stigmatizing attitudes in Dutch health professionals that showed a modest positive attitude toward psychiatry in GPs, mental healthcare professionals and forensic psychiatric professionals (Gras et al. 2015). Thus, although our results are in line with previous research, the use of different questionnaires makes it difficult to evaluate whether and how the level of stigmatizing attitudes toward mental illness in YFC physicians compares to other Dutch healthcare professionals.

Although previous research and guidelines suggest that the Dutch general population has a lower acceptance of mental illness (Beldie et al. 2012), there are not enough studies to substantiate this statement. The trend is visible in, for example, the renaming of an infant autism screening questionnaire omitting the word autism (Van Berckelaer-Onnes et al. 2015). And while previously little attention was paid to reducing stigmatization of mental illness in the Netherlands (Van Weeghel 2005), this has increased in recent years (Netwerk Kwaliteitsontwikkeling GGZ 2017). The trend toward less stigmatization is also evident amongst Dutch preventive care workers. During working groups that were part of our course development process, several YFC physicians stated they were reluctant to attach psychiatric “labels” to children. This is in line with skepticism of the DSM-5 diagnostic system, which includes issues on the potentially harmful effect of receiving a stigmatizing diagnosis (McGorry and van Os 2013). Thus, although there is a visible positive trend to less stigmatizing attitudes towards mental illness in the Netherlands, there are some signs of stigma in Dutch child preventive healthcare services.

The possible effect of educational and professional components on the level of stigmatizing attitudes toward mental illness (Smith and Cashwell 2010)—and the absence of similar studies to our current study—makes it hard to compare the level of stigmatizing attitudes in Dutch YFC physicians to those in healthcare professionals in different countries.

We found no association between ASD knowledge and stigmatizing attitudes toward mental illness after adjusting for age, work experience and income level. Although, in general, training and education have been stated to reduce stigmatizing attitudes toward mental illness (Smith and Cashwell 2010), we found no studies evaluating both ASD knowledge and stigmatizing attitudes.

We also found no association between stigmatizing attitudes and YFC physicians’ age and years of work experience. Previous research showed contradictory results on the association between stigmatizing attitudes and the professional’s age and work experience. While most studies indicate no relationship between stigmatizing attitudes toward mental illness and professionals’ age (Chambers et al. 2010; Kopera et al. 2015; Mosaku and Wallymahmed 2016; Siqueira et al. 2017), other studies found that stigmatizing attitudes both decrease with age (Hansson et al. 2013; Vibha et al. 2008; Winkler et al. 2016) and increase with age (Reavley et al. 2014; Tay et al. 2004). Some studies offer a possible explanation for these conflicting results. First, two large studies (n = 7555, n = 2391) found that stigmatizing attitudes toward mental illness increase with age in healthcare professionals and in the general public (Reavley et al. 2014) but differ per age group in the general public (Högberg et al. 2012), for isolated elements and subscales of stigma. Various levels of stigmatizing attitudes toward mental illness per age group followed a non-linear pattern and were found in a large mixed sample (n = 3010) that included medical doctors and the general population. A bimodal trend of some stigmatizing attitudes toward mental illness during adulthood might explain the absence of correlation between age and stigmatizing attitudes when age is used on a continuous scale (Kopera et al. 2015; Vibha et al. 2008). A bimodal trend would also affect associations when age is divided into two groups based on a mean or median score (Chambers et al. 2010; Hansson et al. 2013), or when age groups are used that are not in line with the trend. Finally, the strong correlation between age and work experience found in professionals indicates that elements associated with work experience (like education and amount of exposure to patients with mental illness) are being measured instead of age.

The effect of work experience-related elements is supported by a study in students and healthcare professionals showing that it was not work experience but training, education and exposure to mental health patients that had a positive effect on reducing stigmatizing attitudes toward mental illness (Smith and Cashwell 2010). One study found a positive effect from work experience on elements of stigma toward mental illness (Mosaku and Wallymahmed 2016) in nurses, physicians and community health workers. However, the use of a sample that included several types of professionals may have affected the results. Different levels of stigmatizing attitudes between healthcare professionals have been found due to workload pressure or a lack of awareness and training about mental health (Reavley et al. 2014), or from work and personal experience (Gras et al. 2015).

Our study shows that knowledge, age and work experience do not affect the level of stigmatizing attitudes toward mental illness in YFC physicians. However, little is known about the factors influencing stigmatizing attitudes toward mental illness. Stigma toward mental illness is seen as pervasive across cultures, societies and professions (Van Brakel 2006), but is also affected by wider social, cultural and professional circumstances (Chambers et al. 2010) and by personal experience with psychiatric patients (Pascucci et al. 2016). Research into such attitudes faces many challenges (Casados 2017) and the complex structure of stigma toward mental illness also complicates the interpretation of results. Our findings show that improvement is also possible. First, the stigmatizing attitudes toward mental illness which are present in Dutch YFC physicians need to be addressed. This can be done by a contact-based educational program developed for YFC physicians. Here, YFC physicians will not only enhance their knowledge, but can also interact with people with mental illness. Previously, a contact-based educational program showed a positive effect by reducing the level of stigma among medical students (Papish et al. 2013). However, others found no effect of an e-learning course on attitudes towards mental illness among psychiatric nurses but this can possibly be explained by the short time span of the study (Pitkänen et al. 2015). Secondly, our findings show that there is a need for YFC educational programs to specifically address YFC physicians specific ASD knowledge. The Live Online educational program developed for this study ‘*Detection of Autism Spectrum Disorders in Children Aged 4–6 Years by Youth & Family Centre Physicians’* addresses physicians specific ASD knowledge on the topics; early detection, red flags of ASD, early detection during consultation and communicating with parents/children and referring. Post and follow-up results of our study will have to demonstrate the effect of the educational program on the level of ASD knowledge of YFC physicians as well as the level of perceived competence, stigma and the number of potential ASD referrals. These studies emphasize the potential relevance of training preventive health workers to optimize the national healthcare systems for the early detection of ASD. However, international studies are needed to evaluate the effect of professionals’ stigmatizing attitudes toward mental illness, and the factors influencing such attitudes, on their early detection of autism in childhood.

### Strengths and Limitations

Our study has several limitations. We used the CAMI to evaluate general stigmatizing attitudes toward mental illness instead of stigma specifically toward autism. We evaluated ASD knowledge with a self-developed questionnaire as we could not identify suitable questionnaires at the start of the study. During the course of this study, the Autism Stigma and Knowledge Questionnaire (ASK-Q) was developed for evaluating both ASD knowledge and stigma and has strong psychometric properties (Harrison et al. 2019; Harrison et al. 2017a). While the CAMI is widely used to assess stigmatizing attitudes toward mental illness in general, it is unknown how the CAMI relates to stigma toward autism. Another limitation is that although our ASD knowledge questionnaire was evaluated by an ASD expert panel and tested by YFC physicians, it showed insufficient psychometric properties. The low internal consistency of our ASD knowledge questionnaire indicates that the total questionnaire is weak as a construct for ASD knowledge, but the high percentage of incorrect answers suggests a gap in ASD knowledge, which is a point for concrete attention.

Another limitation of our study is that 95% of the YFC physicians in our study sample were female. However, the high percentage of females is representative for the total YFC physician population in the Netherlands as currently 93% of them is female (AJN 2019). Also, 60–70% of the students starting medical study in the Netherlands is female, with their percentage increasing each year. Previous studies found higher levels of stigmatizing attitudes in male than in female health professionals (Chambers et al. 2010; Siqueira et al. 2017). Thus, our results regarding stigmatizing beliefs among Dutch YFC physician might be an underestimation when compared to physician samples with a more equal sex distribution. In addition, our study sample containing 95% female YFC physicians seems to reflect the total sample of YFC physicians in the Netherlands. Selection bias by our study seems unlikely as the course was obligatory for all YFC physicians in the Rotterdam municipality. Selection bias by sex could be addressed by explicitly evaluating male physician populations who participate in autism screening of young children. A final limitation of our study is that, due to the small sample size, we could not explore the contribution of ethnicity of the YFC physicians on the level of stigma. As previous research found racial differences among the general population in the level of stigmatizing attitudes toward people with mental illness (Anglin et al. 2006), the exploration of possible ethnic differences in stigma amongst preventive care providers is relevant. Nonetheless, our study has several strengths. We investigated a large group (n = 93) of YFC physicians with a wide range of age and work experience. In addition, we evaluated ASD knowledge and stigmatizing attitudes toward mental illness in the same population at the same time, which has not been done before. Finally, we evaluated the association between ASD knowledge and stigmatizing attitudes toward mental illness while adjusting for demographic variables like age and work experience.

## Conclusion

Autism knowledge and stigmatizing attitudes held toward mental illness are points requiring attention in Dutch physicians screening the general child population for ASD. Our study emphasizes the relevance of ASD training for professionals. Future research should evaluate the effect of ASD knowledge and stigmatizing attitudes toward mental illness on the early detection of autism cases in preventive care providers.

## Electronic supplementary material

Below is the link to the electronic supplementary material.

Supplementary file1 (DOC 60 kb)

## Footnotes

1 Live Online Learning was provided by Learning Connected, an online classroom where participants have real-time aural and visual contact with their teacher.

2 Translated from the Dutch: Kennis en Signalering Autismspectumstoornissen-Jeugdartsen, VKSA-J.
