# Supplementary material for: Novel Insights into Autism Knowledge and Stigmatizing Attitudes Toward Mental Illness in Dutch Youth and Family Center Physicians
Source: Community Ment Health J. 2020 Feb 11;56(7):1318–30. doi: 10.1007/s10597-020-00568-w (PMC7434787; doi:10.1007/s10597-020-00568-w)
Supplement: Supplementary file 1 — Supplementary file1 (DOC 60 kb) [file 10597_2020_568_MOESM1_ESM.doc]

Online Resource

**OR1.** Autism Spectrum Disorder Knowledge Questionnaire – Physicians’ edition (AKQ-P)*

M. van ´t Hof, W.A. Ester & M. Deen

December 2015

**Note: Correct answers are given in bold**

1. Do children with autism have delayed speech development?

1. *Yes*
2. *No*
3. ***Sometimes***

2. True or false, children with autism find it easy to imagine something that is not there, such as what will happen next week.

1. *Right*
2. ***Wrong***

3. Children with autism could experience problems with:

- 1. *Processing sensory experiences, like sounds and odors*

1. *Understanding spoken language*
2. *Use of spoken language*
3. ***All three (a, b and c)***
4. *None of the above*

4. True or false, it is easy for children with autism to understand what others’ mean while communicating.

- 1. *Right*
  2. ***Wrong***

5. Is there a cure for autism?

1. *Yes*
2. ***No***

6. Research shows a link between autism and:

1. *Hereditary factors*
2. *Environmental factors*
3. *Neither hereditary nor environmental factors*
4. ***Both hereditary and environmental factors***

7. True or false, a child with autism does not need social contact.

- 1. *Right*
  2. *Wrong*
  3. ***This varies per child***

8. True or false, research shows that interventions aimed at parents, such as parent training, can have a positive influence on the development of children with autism.

1. ***Right***
2. *Wrong*

9. True or false, most children with autism have special interests or movements.

1. ***Right***
2. *Wrong*

10. Which of the following statements about autism are true?

1. *People can grow out of autism*
2. *Autism is only diagnosed in children*
3. *Both are true*
4. ***Both are false***

11. True or false, children with autism always find it uncomfortable when plans are changed suddenly.

1. ***Right***
2. *Wrong*

12. True or false, autism is diagnosed evenly across all ethnic groups and income groups?

1. *Yes*
2. *No*
3. *It is evenly diagnosed in all ethnic groups, but not in all income groups*
4. ***It is evenly diagnosed in all income groups, but not in all ethnic groups***

13. Which of the following are risk factors for the development of autism?

1. *Hereditary factors*
2. *Parenting features and/or upbringing*
3. *Certain syndromes like: Fragile X syndrome and Down syndrome*
4. *A, b and c*
5. ***Only a and c***

14. True or false, the majority of the children with autism have intellectual disability.

1. *Right*
2. ***Wrong***

15. There are … with an autism diagnosis.

1. ***4 times as many boys as girls***
2. *2 times as many boys as girls*
3. *4 times as many girls as boys*
4. *2 times as many girls as boys*
5. *As many boys as girls*

16. True or false, children with autism do not show attachment, not even to their parents.

1. *Right*
2. *Wrong*
3. ***This varies per child***

17. How prevalent is autism? It is found in:

1. *About 1 in 10 children*
2. ***About 1 in 100 children***
3. *About 1 in 300 children*
4. *About 1 in 1,000 children*

18. Which of the following statements is true?

1. *Autism was discovered in the past 20 years*
2. ***Autism was recognized clinically for the first time in the 1940s***
3. *Autism is becoming an epidemic*

19. What causes autism?

1. *Autism is caused by problems in education*
2. *Autism is caused by a combination of toxic substances from the environment and genetic elements*
3. ***Autism is caused by heritability and environmental factors***

20. True or false, vaccinations play a role in the development of autism.

1. *Right*
2. ***Wrong***

21. How is Autism Spectrum Disorder specified in the DSM-5?

*1. Impairment in social communication and social interaction*

*2. Limited, repetitive behavioral patterns, interests or activities*

*3. Delay in or abnormal functioning in at least one of the following areas starting*

*before the third year: (1) social interactions, (2) language, as used in social communication, or (3)*

*symbolic or imaginative play*

1. ***1 and 2***
2. *1 and 3*
3. *All three*

22. Which of the following statements about the early detection of autism are true?

1. *The signs of autism are only visible after 3 years of age*
2. ***The goal of early detection (0-6 years) of autism is to get children and parents in a treatment program earlier, with the aim of optimizing development of the child with autism***
3. *Both statements are true*
4. *Both statements are false*

23. Which of the following statements about intervention are true?

1. *Early interventions don’t show positive results*
2. *Starting the treatment of early childhood autism will create more healthcare and social costs in the long run*
3. *Both statements are right*
4. ***Both statements are wrong***

24. Which of the following statements are true?

1. *People with autism always have language problems*
2. *People with autism always have speech problems*
3. ***People with autism always have communication problems***
4. *All three are true*
5. *None of the above statements are true*

25. Which of the following statements about the possible signs of autism are true?

1. ***The child doesn’t seem to hear when others talk to him***
2. *The child tends to seek quarrels with other children*
3. *Both a and b*
4. *None of the above*

26. Which of the following statements may describe signs of autism?

1. *Speaks with abnormal tone or voice, or with a strange rhythm or pitch*
2. *Uses facial expressions that do not match what is being said*
3. *Sits on a stranger’s lap quickly*
4. ***Both a and b***
5. *All three (a, b and c)*

27. Which of the following statements may describe signs of autism?

1. *Intentionally hurts animals*
2. *Has difficulty adapting to changes in schedule or environment*
3. *Seems to have tantrums out of the blue*
4. *Both a and b*
5. *Both a and c*
6. ***Both b and c***

28. Which of the following statements may describe signs of autism?

1. *Annoys other children intentionally*
2. ***Often take things quite literally (e.g. “take your coat” and the child takes the coat but without wearing it)***
3. *Both a and b*
4. *None of the above*

29. Which of the following statements about autism are true?

1. *Autism manifests itself the same in boys and girls*
2. *Autism manifests itself similarly in children with learning difficulties as well as in children with a high IQ*
3. *When a child can make eye contact, there is no autism*
4. *A and b are right*
5. *All of the above statements are true*
6. ***All of the above statements are wrong***

30. What is recommended when parents say they are worried that their child may have autism?

1. *Always advise them to wait and see if the child outgrows the problem by itself*
2. ***Provide information about places they can go to with questions, and for advice and information***
3. *None of the above*
4. *Both a and b*

31. What is a common comorbidity in autism?

1. *Sleeping problems*
2. *Gastrointestinal complaints*
3. *Eating problems*
4. ***All three (a, b and c)***
5. *None of the above*

32. What is a common syndrome in autism?

1. *Neurofibromatosis*
2. *Tuberous sclerosis*
3. *Fragile X syndrome*
4. ***All three (a, b and c)***
5. *None of the above*

Thank you for your cooperation!

-----------------------------------------------------------------------------------------------------------------

^*^Translated from the Dutch. Autisme Spectrum Stoornis Kennis Vragenlijst – Jeugdartsen (AKV-J)

Please reference as:

Van ´t Hof, M., Ester, W.A. & Deen, M. (2015) *Autism Spectrum Disorder Knowledge Questionnaire – physicians’ edition (AKQ-P).* Rotterdam, The Netherlands: Sarr Expert Centre for Autism, Lucertis Child and Adolescent Psychiatry, Parnassia Psychiatric Institute.
